# A review of the business case for workforce nutrition initiatives

**DOI:** 10.3389/fpubh.2025.1592601

**Published:** 2025-06-25

**Authors:** Evert-jan Quak, Ayako Ebata, Inka Barnett

**Affiliations:** Institute of Development Studies, Brighton, United Kingdom

**Keywords:** work, nutrition, malnutrition, business, cost savings, absenteeism, productivity, low-and middle-income countries

## Abstract

Undernutrition and malnutrition remain persistent challenges in low-and middle-income countries (LMICs), especially among workers in labour-intensive sectors. Workplace nutrition programmes (WNPs) have shown promising health benefits, but evidence on their business impact remains scarce—particularly in LMIC contexts. This review examines whether WNPs generate measurable business outcomes that could incentivise employer investment. Using a structured literature review (SLR) approach, we systematically analysed 24 relevant studies—10 systematic reviews and 14 empirical papers. Search terms targeted nutrition-related workplace interventions and business outcomes, including productivity, absenteeism, and return on investment. Searches were conducted across Scopus, ScienceDirect, Google Scholar, and grey literature sources. Studies were included if they assessed business-related outcomes of health or wellness interventions with nutrition components. Only four studies were based in LMICs; the remaining 20 were from high-income countries (HICs), underscoring a major evidence gap. Despite this, two main impact pathways emerged: (1) healthier diets improve workers’ concentration and energy, reducing absenteeism and saving costs; and (2) improved nutrition enhances motivation, productivity, and work quality, which may increase sales and revenue. The first pathway is more relevant to skilled workers who are harder to replace, unlike the easily replaceable labour force common in many LMIC industries. In the second pathway, while improved nutrition may boost productivity, structural barriers—such as limited bargaining power in global supply chains—can prevent these gains from leading to better pay for workers. This review outlines key pathways through which improved worker nutrition may benefit businesses and identifies critical gaps in the evidence. It also proposes outcome indicators relevant to private sector stakeholders in LMICs, helping to guide future empirical research.

## 1 Introduction

Globally, 2.8 billion people—35% of the population and over 70% in low-income countries—cannot afford a healthy diet (1). Malnutrition and undernutrition have lasting negative effects, both for households through intergenerational health impacts (2) and for economies at large (3). The burden of poor nutrition is especially severe among workers in labour-intensive, low-wage sectors in low-and middle-income countries (LMICs) (4).

Given that 60% of the global workforce spends one-third of their time at work (5, 6), the workplace is a strategic setting to address malnutrition. Evidence shows that workplace health and nutrition programmes (WNPs) can improve worker health in LMICs (7). However, the link between improved health outcomes and business benefits remains underexplored, especially in LMIC settings. Most studies to date focussed on high-income countries (HICs) (8–11).

This is a critical gap as business are more likely to invest in WNPs when there is evidence of financial returns, such as increased productivity or positive return on investment (ROI) (12–15).

This review examines whether workplace-based nutrition interventions generate business benefits in LMICs, particularly in labour-intensive sectors—such as agriculture, call centres, electronics, and garments. It focuses on health and nutrition outcomes among workers who are often at high risk of undernutrition and explores whether there is a compelling business case for investment in these settings (7). Previous reviews (16) have linked workers’ health and nutrition to business outcomes in HICs (8, 17). LMICs differ significantly in terms of workforce characteristics, economic structures, and health baselines. For example, while HICs often deal with obesity and non-communicable diseases (NCDs) (18), low-skilled workers in LMICs are (still) more likely to suffer from undernutrition and micronutrient deficiencies (19).

Therefore, this review examines the available evidence on WNPs in LMICs but also considers how research from HICs can inform a research agenda tailored to LMIC contexts. The economic context, the specific characteristics of the workforce and their nutritional baseline are distinct between LMICs and HICs (16). In HICs (e.g., the United States and United Kingdom), may invest in workforce health as a means to reduce health care costs (20), employees in LMICs would not see such financial benefits. This is because employers would not pay for employee’s health insurance. Similarly, as workforce in LMICs tends to be dominated by inexpensive and unskilled work in labour-intensive and relatively low-technology industries—e.g. textiles and agribusiness—the cost of replacing labour is low, making investment in the current workforce unattractive to employers (21).

Finally, WNPs alone are unlikely to address under-and malnutrition in the LMICs’ context with high informality of labour force, gender inequality, and overall low wage (22, 23). Nonetheless, WNPs may still offer a cost-effective way to improve health outcomes for workers, especially where nutrition baselines are low and the potential for rapid gains is high (24).

In this paper, we define WNPs as part of employer-sponsored programmes aimed at improving employee (and sometimes also their families) wellbeing and productivity (25). These may include nutritious food provision, health screenings, nutrition education and awareness raising as well as financial incentives for behaviour change (26–29).

The article proceeds as follows: Section 2 outlines the review methodology, followed by a synthesis of evidence on business benefits in Section 3. Section 4 introduces a conceptual framework, which is then applied to LMIC contexts in Section 5. The article concludes with recommendations for research and business practice.

## 2 Methodologies and approaches

We employed the Structured Literature Review (SLR) method as discussed by Paré and Kitsiou (30) and Littell (31). This method is a rigorous, transparent, and replicable method for reviewing existing literature. It is especially useful in identifying, assessing and synthesising the most influential and relevant research on a given topic. This method was chosen due to its greater flexibility compared to the PRISMA protocol, particularly in accommodating iterative strategies such as snowball sampling and the inclusion of grey literature. Despite this flexibility, the SLR approach maintains systematic and transparent selection procedures that emphasise the identification of high-impact research. This methodology is especially suited to the objective of developing a conceptual framework grounded in existing empirical evidence, aligning with the recommendations of Snyder (32). Furthermore, previous evaluations suggest that structured reviews may produce conclusions comparable to those derived from systematic reviews, indicating their validity in synthesising research findings (33, 34). The initial literature search focussed on the business and management scholarship, but was later expanded to include health literature due to a limited number of relevant studies pertaining to WNPs in the original domain. Search engines used are Scopus, ScienceDirect and Google Scholar.

We also searched for grey literature using the Google search engine and identified eight relevant reports. However, these were high-level policy documents that lacked empirical data or evidence on the business outcomes of workplace-based nutrition interventions. Notably, we found no evaluations of specific WNPs implemented in LMICs—whether by NGOs or private-sector actors. This supports the view that such programmes are limited in LMICs and suggests that businesses may be reluctant to share sensitive information related to productivity and other outcomes, often prioritising health impacts instead. Consequently, we focused our analysis on the available academic sources.

The reviewed literature is divided according to two categories: (1) systematic reviews on WNP-related business outcomes (see Table 1) and (2) academic empirical studies on the business outcomes of relevant workforce interventions (see Table 2). The search terms were structured in three steps and used in different combinations. The first set of search terms were chosen to elicit studies that evaluate health and nutrition programmes at workplaces. We combined the following terms: “programme,” “interventions,” “employee,” “workforce,” “workplace,” “worksite,” “nutrition,” “micronutrients,” “nutrition education,” “wellness,” “health.” The second set of search terms that we combined with the first one had the aim to determine the outcome indicators. We used the following terms: “business outcomes,” “workplace outcomes,” “economic outcomes,” “productivity,” “business output,” “cost savings,” “absenteeism,” “presenteeism,” “medical costs,” “job satisfaction,” “staff turnover.” Finally, we added search terms related to geographical scope (e.g., “LMICs,” “developing countries,” “Africa”), sectors (e.g., “manufacturing,” “garments,” “agriculture,” “agrifood”) and topic relevance (e.g., “business case,” “return on investment”). Different combinations of these search terms were used as was some form of snowballing.

**Table 1:** Overview of reviewed systematic reviews.

Reference	Intervention	Region	About the study
Anderson et al. (76).	Workforce Wellness Programmes (including nutrition elements)	HICs	Review focus on programmes that tackle overweigh. No ROI as business benefits were rarely measured in the reviewed studies.
Baicker et al. (35).	Workforce Wellness Programmes (including nutrition elements)	HICs	Review of measuring business benefits for workforce health programmes. It found that medical costs fall by about US$3.27 for every dollar spent on the programmes and that absenteeism costs fall by about US$2.73 for every dollar spent.
Baxter et al. (17).	Workforce Health Programmes (including nutrition elements)	HICs	Review of measuring ROI for workforce health programmes. Twenty of the 51 reviewed studies relate to direct outcomes only, mainly based on medical claims and records. The weighted mean ROI was 1.38.
Grimani et al. (8)	Workforce Wellness Programmes (including nutrition elements)	HICs	Review of the evidence on the effectiveness of workplace interventions to address issues of fitness and nutrition. It mentions evidence of reduced absenteeism and some forms of improved productivity, such as presenteeism. No ROI.
Haas & Brownlie (49)	Workplace Nutrition Programmes	HICs & LMICs	Review of the impact of iron supplements on workforce outcomes. Iron deficiency reduces work productivity observed in field studies which is likely due to anaemia and reduced oxygen transport. The causality ratings on productivity levels tended to be lower than ratings for the non-economic outcomes. No ROI.
Jensen (47).	Workplace Nutrition Programmes	HICs	Review of workforce nutrition programmes measuring economic and productivity outcomes. The majority of studies provide evidence for positive productivity effects of worksite interventions. No ROI in most studies.
Lerner et al. (9)	Workplace Health Promotion Programmes	HICs	Review of the economic impact of worker health promotion programmes. The evidence is often of low quality and economic impact is limited and inconsistent. 8 studies claim cost or productivity savings. ROI cannot be measured, according to the authors, because there are too few methodologically strong studies.
Marcus et al. (10)	Micronutrients for the workforce	HICs & LMICs	Review of anaemia issues in labour intensive and non-labour intensive sectors in LMICs. There is strong evidence that anaemia negatively impacts occupational performance and that therapeutic iron interventions can yield substantial productivity gains. No ROI.
Osilla et al. (36)	Workforce Wellness Programmes (including nutrition elements)	HICs	Review of business outcomes for workforce wellness programmes. 8 out of 33 reviewed studies measured health and medical cost savings, with 5 of the 8 studies conducted ROI analyses (between US$1.65 and US$6.00 saved for every dollar invested). Only 4 studies measured absenteeism, which found significant effects, however, measured differently (ROI of US$15.60 per dollar spent).
Schliemann & Woodside (11).	Workforce Wellness Programmes (including nutrition elements)	HICs	Review of business outcomes for workforce wellness programmes. 8 studies estimated work-related outcomes, i.e., productivity, return on investment, health-care costs and sickness/absenteeism. Only two reviews reported a clear positive change in work-related outcomes as a result of a dietary intervention.

Source: Authors’ own (2023).

**Table 2:** Overview of reviewed studies on specific interventions.

Reference	Country	Sector	Company	Intervention	Indicators	ROI
Berry et al. (18)	USA	Multiple	Multiple companies	Workplace Wellness Programme	Health cost savings, productivity, voluntary turnover rate	Yes
Caloyeras et al. (78)	USA	Food	One company	Workplace Wellness Programme	Healthcare cost savings	Yes
Gopaldas and Gujral (48)	India	Agriculture	One tea plantation	Workforce Nutrition Programme	Productivity of women workers.	No
Gowrisankaran et al. (77)	USA	Health	One company	Workplace Wellness Programme	Healthcare cost savings	No
Gubler et al. (43)	USA	Laundry	Multiple companies	Workplace Wellness Programme	Productivity	Yes
Jones et al. (40)	USA	Higher education	One company	Workplace Wellness Programme	Healthcare cost savings, absenteeism, productivity	Yes
Kumar et al. Kumar et al. (44)	USA	Security	One company	Workforce Nutrition Programme	Productivity	No
Lee et al. (39)	UK	Health	One company	Workplace Wellness Programme	Absenteeism (days per capita), voluntary turnover rate	No
Marshall (51)	USA	Hospitality	One company	Workplace Wellness Programme	Job satisfaction	No
Merrill et al. (28)	USA	Public services	One company	Workplace Wellness Programme	Health care costs	Yes
Milani & Lavie (37)	USA	Manu-facturing	One company	Workplace Wellness Programme	Medical claim costs	Yes
Plotnikoff et al. (45)	USA	Health	One company	Workforce Nutrition Programme	Presenteeism	No
Qaisar et al. (46)	Pakistan	Public services	Multiple companies	Workplace Wellness Programme	Productivity (employee and firm productivity)	No
Song and Baicker (20)	USA	Retail	One company	Workplace Wellness Programme	Healthcare costs, Absenteeism, Work performance	No

Source: Authors’ own (2023).

The lead author was responsible for the search and initial selection of the literature, while the co-authors checked on this process independently by using the search terms and verifying relevant sources were included for analysis. We included studies if they reported business outcomes of WNPs, or workforce health or wellness programmes that include nutrition elements in their interventions. This search generated 10 systematic reviews relevant for WNPs and 14 empirical studies researching several outcomes of programmes targeted at the workforce in which nutrition was a component. This generated 20 studies from HICs and 4 from LMICs. We did not exclude evidence from HICs because of the lack of studies from LMICs. Although this is a limitation, we can learn from this evidence and discuss how they can be useful to identify impact pathways for the context of LMICs. In addition, only 6 studies addressed nutrition-related interventions specifically while the remaining studies examined a broader set of wellness and workforce health programmes with elements of nutrition included. Because of the limited availability of empirical evidence on the business case for WNPs a narrower scope (e.g., agriculture sector) would not have yielded sufficient number of studies to review.

Because the selected literature made some suggestions about the relevance of job satisfaction and business reputation, but without providing evidence, we supplemented with additional literature from business and management literature on these topics. This gave us better insights how these two specific outcome indicators can be included in our conceptual framework for their contributions to the business case.

## 3 Findings

Selected studies largely focus on two specific business outcomes: (1) cost saving; and (2) sales and revenue increase. Cost savings relate to reduced healthcare costs (12 studies), reduced sickness absenteeism (8 studies), and voluntary reduced staff turnover (4 studies). Healthcare costs can be reduced through decrease in health insurance premiums (specifically in contexts where employers pay for employee health insurance such as in the US). Reduced staff turnover leads to decreased need to replace staff, and therefore reduced cost of recruitment of temporary staff. Companies’ revenue may increase due to workforce interventions increasing labour productivity, reducing presenteeism, or improving work performances. Examples of measures that can be used to measure productivity include change in the number of output units produced per worker in a specific time, reduced mistakes, increased quality of work outputs. In the next sub-sections, we discuss the evidence for the business outcomes in detail.

### 3.1 Cost saving

The first pathway to reduce cost is through the annual or monthly cost reduction per worker due to reduced healthcare coverage costs and workers’ compensation, including claimed costs and legal fees. Particularly the US literature on workforce initiatives has a clear focus on reducing health insurance cost as a business outcome because American companies pay for employee health coverage, which is different for more public health systems, like in Europe. The evidence from systematic reviews shows that workforce interventions in such context can reduce healthcare costs (17, 35, 36). Milani and Lavie (37) measured that workforce interventions by a US-based manufacturing company reduced the average employee annual health claim costs by 48% for the 12 months after the intervention, whereas the costs for employees’ who did not participate in the programme remained unchanged. Merrill et al. (28) measured for a large local public service provider in the US that the cost savings in lower prescription drug and medical costs was US$3,568,837 in 5 years after the workforce intervention. On the other hand, the study by Song and Baicker (20) did not find strong empirical evidence for reduced healthcare costs for a workforce health and nutrition programme implemented by a large warehouse retail company in the US. They recognise cost saving outcomes from health-related interventions while cautioning that causal relationship is difficult to establish because of the different populations, geographies and employment settings.

The second pathway to reduce cost is through reduced absence from work related to sickness, which is defined as the average number of reduced absences from sickness per worker per month or year (38). When workers are frequently absent, businesses must find temporary replacements (sometimes for specialised tasks), which can increase the workload for others, disrupt the remaining workforce and workflow, cause resentment among employees who feel the absences are not properly addressed, lower overall morale, and risk fostering a culture of frequent absenteeism throughout the organisation. The evidence from selected systematic literature reviews shows that companies have managed to reduce the costs of absenteeism after implementing workforce health and nutrition programmes (8, 35, 36, 39). Lee et al. (39) mention that workforce health and nutrition programmes can reduce sickness absence by between 25 and 30% within 4 years. However, Song and Baicker (20) only found minimum cost savings for reducing absenteeism after a retail company started their workforce health and nutrition interventions. In their study workers were absent for a mean of 2.5% of scheduled hours in the treatment group versus 2.6% in the control group.

The third pathway towards cost reduction is through reduced voluntary staff turnover. This is based on the reduction in annual average turnover rate during the period of the intervention. The cost of replacing an employee typically includes recruitment expenses and can also account for reduced productivity, management time, and training costs during the initial phase. Although only two of the reviewed studies have looked explicitly at the cost savings of voluntary staff turnover (18, 39), the evidence suggests that such savings are achievable. For example, organisations with highly effective workforce health and nutrition programmes report lower voluntary attrition than those with low effectiveness of such programmes (18).

Most studies that measured the ROI did this only for cost saving indicators. The ROI from reduced healthcare costs ranges from US$1.65 (36), US$1.38 (17), US$3.27 (35) to US$6.00 (36). Naturally, ROI measured in countries where employers cover healthcare costs for employees (e.g., the US) is higher than other contexts. The overall healthcare provision and financing also influence the cost—and therefore cost saving—for the employers (e.g., publicly funded healthcare such as in the case of the United Kingdom in comparison to the US where health insurance is covered by the employers). For absenteeism, Baicker et al. (35) systematic review reports an average ROI of US$2.73 saved for each dollar spent on workforce programmes. One of the four studies reviewed on absenteeism by Osilla et al. (36) measured an ROI of US$15.60 per dollar spent. However, the study by Jones et al. (40) for a workforce health and nutrition programme in Higher Education only found US$0.60 saved for each dollar spent.

Overall, ROIs cannot be easily compared to each other as each programme is implemented differently in different contexts and timeframes (17). As a result, most systematic reviews on the ROI of workforce health and nutrition programmes highlight mixed results (11, 12, 17, 36). Generally speaking, however, single-component interventions that provide only one type of activity, like providing food only, or health checks only, are most likely to have a negative ROI (17) and more comprehensive programmes are more likely to result in positive ROI (7). Also, the more employees participate in the same programme, the higher the ROI is for the implemented business (17).

### 3.2 Increased sales and revenue

The reviewed literature shows that the impact pathway for increased sales and revenue are measured in two ways, by reduced presenteeism and increased labour productivity. The two are interrelated, and as such often studied together, because improved employee productivity due to a workforce intervention may be the result of reduced presenteeism. Low presenteeism means being physically present at work and improving the workload due to being fully alert and energised and capable of work. Loeppke et al. (41) and Collins et al. (42) argue that high incidence of presenteeism costs businesses financially more than absenteeism. In general, the assessed literature shows positive outcomes on presenteeism often in combination with labour productivity gains (43–46).

Plotnikoff et al. (45) find that vitamin D supplements given to workers in healthcare increased productivity due to presenteeism. Workers with the highest vitamin D levels notified better work performances. Gubler et al. (43) also find evidence of productivity improvements of workers in a laundry business (10% labour productivity gains) due to workforce health and nutrition interventions which were based on reduced presenteeism, related to improved capabilities and motivations. Qaisar et al. (46) measured perceived changes in presenteeism and productivity at the managerial level for multiple businesses in Lahore, Pakistan. They argue that there is a correlation between improved capabilities, stress control, and creativity of the workforce due to participation in workforce-related health and nutrition programmes on labour productivity and organisational productivity. The latter relates to higher or improved outputs, quality, speed and flexibility within the organisations.

However, the literature argues about methodologies used to measure the value of presenteeism with most studies using self-reported data from participants through questionnaires instead of available business data (8, 17, 35, 47). The general perception from these studies is that measuring perceived changes in presenteeism and productivity are subjective and therefore provide less rigour evidence for understanding the business case.

On the other hand, reviewed studies in LMICs also measure labour productivity by increased outputs per worker—mostly in agriculture (e.g., kilogramme of picked tea per worker per day) (10, 48). Productivity as an output level has been measured most often in combination with interventions that target micronutrient deficiencies, mostly iron deficiency related anaemia, in LMICs. Marcus et al. (10) shows that these studies use different methods, mainly cross-sectional studies, placebo-controlled trials, and mixed methods of both. Of the nine reviewed studies by Marcus et al. (10), most show some positive outcomes, with only one finding no significant impacts. Such studies make use of average output improvements following treatment or by comparing the anaemic and non-anaemic workers (10, 45, 48, 49). The highest productivity improvements can be made for the groups with severe and medium high iron deficiencies (48, 49), indicating the importance of baseline measures to understand productivity outcomes.

Importantly, productivity outcomes from workforce interventions are highly dependent on the characteristics of the workers, work tasks, as well as sectors. For example, more automated and mechanised work might reduce the productivity impact of these interventions (10). Likewise, if fewer workers are needed to produce the same volumes (48), eventually this should translate to higher payment to workers.

### 3.3 Structural barriers and contextual factors

The selected studies (both empirical studies and systematic reviews) take into account work arrangements (e.g., full-time work), firm characteristics (e.g., large firms), and nutritional baseline as factors that influence on business outcomes of these interventions. Although the literature acknowledges that large firms are overrepresented, they assume that smaller sized businesses can struggle to implement more comprehensive WNPs, due to limited capacities and resources (17). The literature also mentions that employees with specific work tasks will benefit more than others, such as working remotely or based at various worksites, which will limit accessibility of certain workers to these programmes (17, 35). Furthermore, the programmes engage differently with casual, seasonal, or part-time workers compared to full-time workers (39). Finally, there is to some extent the recognition that out-of-work behaviours of workers can impact on the outcomes (20).

Wider contextual factors are hardly mentioned in the selected studies. These could relate to structural barriers, for example in the value chain governance systems to raise wages, or enabling factors, for example subsidies on providing healthier meals. For example, US regulations such as the Workforce Investment Disclosure Act of 2021 require public companies to report on human capital metrics—many of which pertain to employee well-being. This regulatory landscape is expected to grow, increasingly pushing organisations to take measurable steps in supporting their workforce. The absence of evidence on how wider contextual factors influence the business outcomes of WNPs may stem from a lack of comparative studies across countries, sectors, value chains, and political contexts.

## 4 Conceptualisation of impact pathways

**Figure 1:**
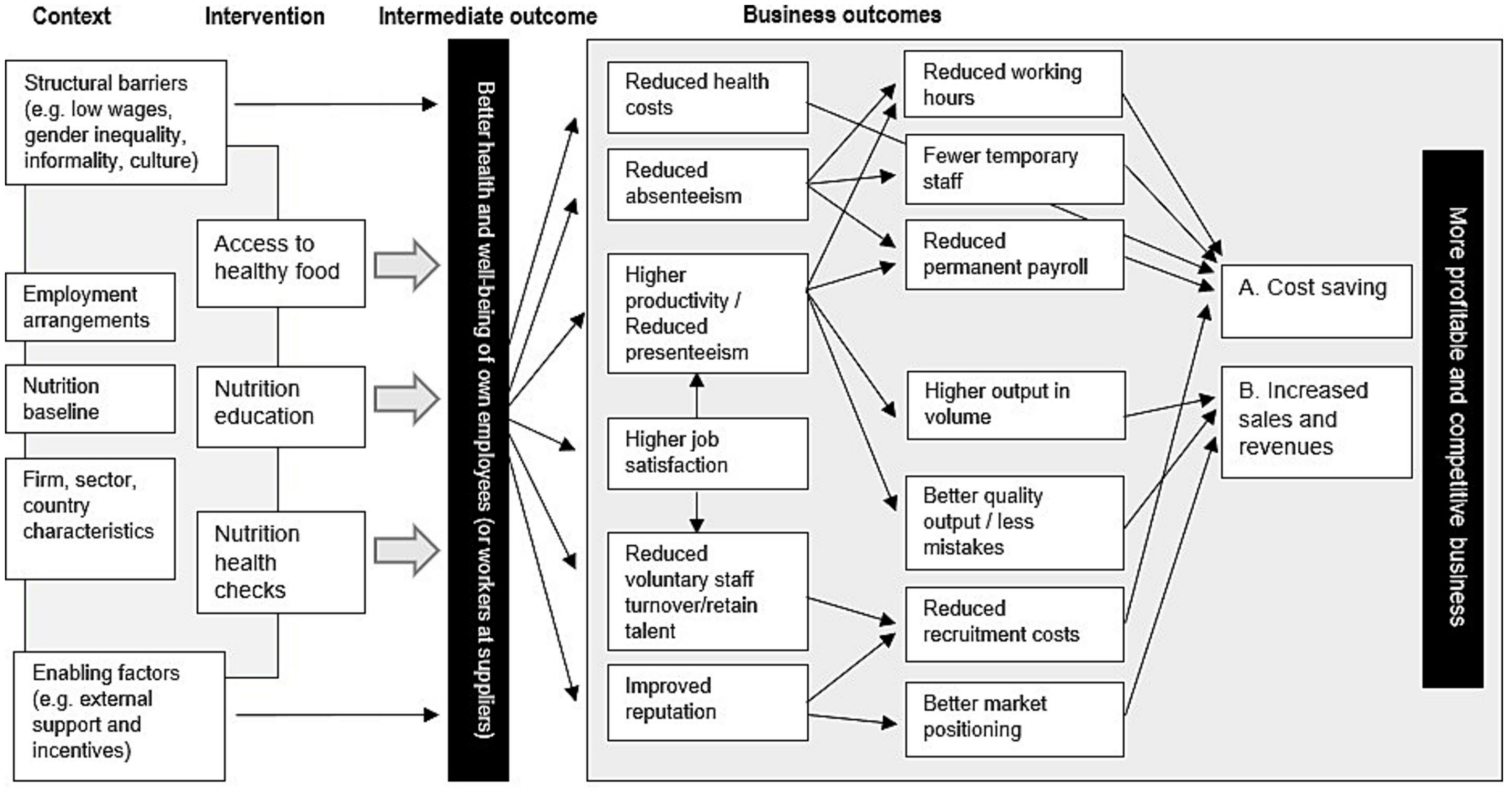
Impact pathways for linking business benefits with WNPs. Image created by the authors.

Based on the summarised evidence, which highly rely on evidence from HICs, we now visualise impact pathways that link workforce-based interventions on nutrition and health on business outcomes in Figure 1. We also discuss later that the application of HICs findings to conceptualise impact pathways in LMICs contexts should be considered speculative due to the distinct settings in which these programmes are implemented. Figure 1 shows that intervention (i.e., workforce nutrition programmes) is situated in a particular setting, which leads to intermediate outcome (i.e., improved health and well-being of employees). These health outcomes among employees lead to the business outcomes discussed above—cost saving and increased sales and revenue—which ultimately benefits the implementing businesses.

Upon deciding to implement a workforce-related health and nutrition programme, each enterprise needs to first determine what intervention(s) are required to tackle employee nutrition issues at baseline. For this, the enterprise needs a good understanding of the specific employment arrangements and conditions for participation in the WNP, which relate to the employee nutrition baseline, as well as the characteristics of the job and sector they work in such as physicality, seasonality, or location of the work (8, 47).

Wider contextual factors, such as structural barriers and enabling factors, need to be considered for WNPs because they can enable and impede impact pathways. Structural barriers and enabling factors are context specific and different for each country and sector. The selected literature did not specifically highlight these factors, but studies that assess impact pathways and theory of change (50) for specific interventions recommend the inclusion of them. For example, low wages, gender inequality, and job informality can impede the motivation of businesses to establish such interventions. On the other hand, potential institutional support by governments, donors or lead firms in the value chain can incentivise certain aspects in design and implementation of these programmes. Therefore, contextual factors are included in the conceptual framework.

In the business outcome box of Figure 1, we include job satisfaction and improved business reputation as an important measure for generating business outcomes through WNPs. This is because, the reviewed literature suggests that improved employee satisfaction can be associated with improved productivity and reduced voluntary staff turnover, as well as the workforce health and nutrition programmes being a recruiting tool in order to attract top talent (39, 47, 51). Employee satisfaction is measured as a level of motivation, loyalty, pride, and intent to stay with the company (51). Marshall (51) concludes that participating in workforce health and nutrition programmes increases and maintains current employees’ extrinsic and intrinsic job satisfaction levels, with spill-over impacts to both cost savings and productivity gains.

Additional search for studies confirms this, although not specifically for workforce-based interventions. Harter et al. (52) showed that employee satisfaction correlates with work-related benefits at the business level (not only individually) through higher productivity, profitability, customer satisfaction–loyalty, and employee turnover. Oswald et al. (53) measure a strong correlation between business outcomes by higher motivation levels. However, the authors note difficulty in establishing a causal relationship and identifying monetary benefits from employee satisfaction.

Based on a meta-analysis of 230 companies, Krekel et al. (54) show a robust correlation between job satisfaction, productivity and firm performance. They state that job satisfaction has a substantial positive correlation with customer loyalty and a substantial negative correlation with staff turnover. Higher customer loyalty and employee productivity, as well as lower staff turnover, are also reflected in higher profitability of business units, as evidenced by a moderately positive correlation between employee satisfaction and profitability. Further, there are some differences between industries: the magnitude of correlation is highest in in finance, followed by retail and services (54). For manufacturing, the authors find that employee satisfaction has the weakest correlation with employee productivity, but the strongest with business unit profitability among all industry sectors. This may be because manufacturing focuses on process efficiency and safety as primary metrics within plants, which relate directly to costs. Therefore, job attitudes are likely to relate to discretionary effort, which affects quality, efficiency and safety within manufacturing plants and teams, possibly explaining the higher correlation between employee satisfaction and business unit profitability in that sector (54).

Another additional component in Figure 1 is improved reputation for generating business benefits from workforce health and nutrition programmes, which was only mentioned as a possible important business benefit for WNPs in the study by Lee et al. (39). While existing evidence does not empirically test the reputational benefits from such programmes, the idea is that workforce health and nutrition programmes help increase the company’s reputation amongst consumers, shareholders, and employees, which leads to financial benefits. To elicit available evidence, we referred to the literature on corporate social responsibility (CSR). The CSR literature suggests that improved corporate image can reduce recruitment costs and can improve profitability particularly in sectors with high visibility in competitive consumer markets (55). Because many companies base their prices on brand image and ‘goodwill’, reputational effects become important measures of success for their market value and profit margins (56). Hence, company’s improved CSR through, for instance, investing in employees’ health, companies are able to showcase their improved productivity, employee satisfaction and engagement, consumer loyalty, and other beneficial outcomes to external stakeholders, which can maximise its corporate image and ultimately competitiveness (57–59).

The inclusion of job satisfaction and reputation as potential pathways is based on general business and CSR literature that require further investigation within the specific context of WNPs.

## 5 Discussing impact pathways in the context of LMICs

While this paper aimed to gather evidence from both HICs and LMICs, our review highlights an evident lack of research in LMICs, linking workforce nutrition and health programmes to business outcomes. The reviewed literature shows that the business outcomes of WNPs depend on the contextual circumstances, such as the baseline nutrition (and related health and wellness) issues in the workforce, which vary significantly per company, sector, and region (16). This implies the importance of explicit focus on LMICs in understanding how nutrition and health interventions in workplaces can influence employee’s health, and in turn business outcomes at the employer level. As malnutrition and labour dynamics are distinct in LMICs than HICs (19, 21, 24), this lack of evidence from LMICs has important implications on the nature of empirical evidence.

First, the dynamics of malnutrition in LMICs is increasingly complex where resource-poor countries, households and individuals are starting to face the ‘double burden of malnutrition’, characterised by the coexistence of undernutrition along with overweight, obesity or diet-related NCDs (60). Such complex realities necessitate looking beyond the current focus on vulnerable population such as women of reproductive ages and young children – i.e. under five and those attending schools (61) – and extending the research focus on ways to improve health and nutrition of working adults. The evidence from WNPs can be linked with evidence from school feeding programmes (62) to ensure long-term nutritional advances into adulthood. However, with hardly any evidence on the business case for such interventions in the context of LMICs, there is no clear incentive for businesses to make these investments in LMICs.

Another implication of the lack of evidence from LMICs is that the indicators assessed to measure business outcomes need to be better tailored to the contexts and challenges in LMICs. Our review shows that indicators that assess different cost savings of workforce interventions are the main target area of research in HICs. However, the reality of cost saving depends on the national health systems and insurance dynamics where, for instance, companies based in the US may see an increased benefit from improving employees’ health as they incur the cost of health insurance for the workforce, unlike those in Europe. These differences in healthcare realities need to be specifically addressed in the context of LMICs to evaluate the business outcomes of WNPs.

Another shortcoming of the reviewed evidence is that studies focusing on HICs tend to be on workforce from tertiary sectors (see Table 2). Such workforce is distinct from the sectors and the kinds of work more common in LMICs (e.g., agrifood, garment and textile, construction and manufacturing sectors) where the nature of work is more labour-intensive and workers tend to be lower-skilled and low paid (63, 64). These workers have the highest nutritional needs (24) as they tend to come from socially and economically marginalised backgrounds. Without empirical evidence, the implications of WNPs for these workers is unclear. On one hand, companies in these sectors in LMICs may achieve less cost reduction because such workers are inexpensive to employ and easier to replace (63). On the other hand, productivity gains and reduced presenteeism, combined with higher job satisfaction can result in higher revenues and sales for businesses in labour-intensive industries through business investments in WNPs (24). Also, paying attention to the difference within a workforce will be particularly crucial in LMICs. For example, nearly two-thirds of wage employment is casual and/or temporal in Bangladesh and India (65). Workers with formal and informal arrangements with a firm will likely have different socio-economic backgrounds as well as access to WNPs. Furthermore, it is relevant that research in the context of LMICs considers structural barriers related to firm characteristics, sectors and regions. Smaller businesses might not be able to invest in comprehensive WNPs, because they do not have sufficient space and capacities, but could benefit from certain elements of interventions (e.g., nutritional education). High informality and casual work arrangements could impede business leaders to invest in these programmes, but some could still be interested if there is clear evidence of shorter-term business benefits.

In general, a legitimate question that needs to be asked, is why (particularly larger companies) do not pay higher wages for their workers for them to be able to purchase healthier food. The low wages paid, particularly in labour-intensive low skilled industries in LMICs are not disputed with the introduction of WNPs. However, potential higher productivity outcomes, as a result of their implementation, should result in positive wage responses by businesses. However, evidence from LMICs show clearly the systemic issues related to the persistence of low wages, such as captive value chain governance, low added values, low bargaining powers, and high supply rate of low-skilled and cheap labour (66–69). These systemic issues keep wages low in LMICs and negatively impact on healthier food purchasing power (70). Low wages also impede directly the impact pathways as presented in the conceptual framework. Business leaders might not be incentivised by low wages to invest in WNPs. Evidence also shows that an increase in income does not always increase healthier and nutritional diets at household level (71). That means that WNPs have the potential to directly improve the nutritional baseline for workers and behavioural change towards healthier diets in the medium to longer-term (7), even when market factors might impede progress in wages (72). However, the business case for doing this through the two conventional pathways might reduce the interest for doing so.

Therefore, the framework includes reputation as indicator for the business case as well, with the acknowledgement that this is mostly relevant for larger businesses. Improved reputation towards wider stakeholders (e.g., buyers, employees) could encourage business leaders to look beyond a pure focus on return on investment or other monetised business outcomes.

A focus on LMICs may elucidate a crucial role played by (international) lead firms in supply chains to (co-) invest in WNPs at their suppliers in LMICs. International lead firms have more financial resources and capacities to invest in such interventions (73), and they are under increased scrutiny to deliver social and ecological outcomes in their home countries (74). Investing in workforce in LMICs may lead to international firms securing supplies by building stronger relationships and creating supplier loyalty (13). While the financial benefits for international firms to invest in workforce health and nutrition in LMICs are currently assumed in the literature (24), there is no empirical evidence to support that such business outcomes exist along global supply chains.

Finally, WNPs should be considered as part of the decent work conditions of the International Labour Organisation (ILO), and ideally should be integrated with broader workers health and wellbeing activities which allow workers not only to have access (and time) at work to nutritional and healthier food options, but also to combine this with access to safe drinking water at the workplace, refreshment areas for workers, and breastfeeding facilities at work, among others (75).

## 6 Conclusion

This paper conducted a structured literature review of 24 studies to understand the business outcomes of health and nutrition workforce interventions. Businesses have an opportunity to improve the health and nutrition of their workforce as workers spend significant amount of time at work. However, business leaders would not be incentivised to invest in such interventions unless there is clear evidence for financial benefits (e.g., positive return on investment, ROI) to the business. This review showed that studies that explored the business case for such interventions find a positive business case for these programmes, particularly for larger companies, although with large differences in study design and in the success ratios. Business outcomes are primarily measured in terms of reduction in healthcare costs, absenteeism and voluntary staff turnover, and increase of productivity. Company reputation and employee job satisfaction are often assumed but rarely measured.

Based on the reviewed evidence, which comes mainly from HICs, two key impact pathways can be identified. First, workers have higher concentration and energy levels due to healthier diets, which reduces sickness absenteeism, needs to employ temporary staff, and healthcare cost. Second, workers feel better at work (motivational) and have higher concentration and energy levels, which improves their productivity and quality of work (e.g., less mistakes and accidents), leading to increased sales and revenues.

However, the review identified a lack of evidence on the business outcomes of WNPs in the context of LMICs. First, and perhaps most importantly, there is a lack of empirical evidence in LMICs. Among the studies we reviewed, 8 out of the 10 systematic reviews, and 12 out of the 14 individual studies addressed workforce programmes exclusively in HICs. This is problematic because the contexts (e.g., employment arrangements, nutrition baseline) in HICs are very different from those in LMICs, and therefore will have different impact on the business benefits of such programmes. Therefore, we recommend a strong focus on evaluating WNPs in LMICs specifically. Empirical evidence from LMICs is vital to understanding the impact pathways; whether and how WNPs may lead to positive business outcomes for companies employing people, particularly in labour-intensive industries in LMICs.

Second, existing evidence does not attribute the difference in business outcomes from workforce-based interventions to the characteristics of workforce, enterprises, and work involved. This is a missed opportunity to generate more generalisable analysis, indicating what works when, under what conditions and for whom. In general, more WNPs need to be assessed in order to understand the pattern regarding, for instance, how different contexts (e.g., countries, national health systems, sectors, firm sizes, etc.), interventions (e.g., those focused on wellness vs. nutrition) and workforce characteristics (e.g., baseline nutrition status, types of work, types of contracts) influence the outcomes of workforce health and nutrition programmes. This also relates to broader systemic issues in LMICs that need to be understood as factors influencing the impact pathways.

Third, reviewed studies measure only a selection of indicators related to cost savings and productivity gains while other business outcomes – such as job satisfaction and reputation – are identified as critical based on anecdotal evidence. Future studies should include such indicators to test and validate their contributions to impact pathways, linking WNPs to the commercial and financial performance of implementing firms in LMICs. Such evidence can not only encourage more businesses to invest in WNPs in LMICs but also assist them in designing effective programmes to achieve both health and business goals.

Finally, existing literature focuses on programmes implemented by the firms that employ their own workers, but not those implemented by international lead firms along the supply chain. International lead firms may be willing to support and invest in workforce health and nutrition programmes for their supply chain workers, for example farmers or workers in garment sector in LMICs.

Addressing these evidence gaps for LMICs is critical to understand how business leaders could invest in WNPs to benefit marginalised people working in labour-intensive industries while benefitting the business. This would give vital evidence that is now lacking on the effectiveness of WNPs in addressing under-and malnutrition in LMICs.
